# Membranes for the Gas/Liquid Phase Separation at Elevated Temperatures: Characterization of the Liquid Entry Pressure

**DOI:** 10.3390/membranes11120907

**Published:** 2021-11-23

**Authors:** Sara Claramunt, Florian Völker, Uta Gerhards, Manfred Kraut, Roland Dittmeyer

**Affiliations:** Institute for Micro Process Engineering (IMVT), Karlsruhe Institute of Technology, 76344 Eggenstein-Leopoldshafen, Germany; uqraf@student.kit.edu (F.V.); uta.gerhards@kit.edu (U.G.); manfred.kraut@kit.edu (M.K.); roland.dittmeyer@kit.edu (R.D.)

**Keywords:** liquid entry pressure, hydrophobic membrane, membrane distillation, steam removal, high temperature

## Abstract

Hydrophobic membranes were characterized at elevated temperatures. Pressure was applied at the feed and permeate side to ensure liquid phase conditions. Within this scope, the applicability of different polymeric and ceramic membranes in terms of liquid entry pressure was studied using water. The Visual Method and the Pressure Step Method were applied for the experimental investigation. The results show the Pressure Step Method to be an early detection method. The tests at higher pressure and temperature conditions using the Pressure Step Method revealed the temperature as being the main factor affecting the liquid entry pressure. Novel LEP data up to 120 °C and 2.5 bar were obtained, which broadens the application range of hydrophobic membranes.

## 1. Introduction

Hydrophobic porous membranes for gas/liquid phase separation can be applied for gas-liquid membrane contactor, pervaporation, and membrane distillation [1]. In the case of membrane distillation, most of the research is performed at ambient up to moderate temperatures (<80 °C and ambient pressure) [2,3].

However, there exists a huge application opportunity in the use of hydrophobic membranes for higher temperature applications, such as in membrane micro-reactors for high-temperature conditions [4]. This is the case, for example, in the intensification of condensation reactions at temperatures higher than 80 °C that are limited by equilibrium [4]. Such high temperatures imply several challenges like the adequacy of a membrane material for the separation purpose and the suitable surface properties to prevent the reaction product from entering the pores [1,4].

The positive effects of increasing the feed temperature on the distilled flux and thermal efficiency of membrane distillation have been already published [5,6]. The exponential relationship between vapor pressure and temperature leads to larger vapor pressure gradients when the temperature is increased [7]. Singh and Sirkar [8,9] verified experimentally the basic feasibility of successful operation at temperatures >80 °C and Luo and Lior [6] made a simulative study of the direct contact membrane distillation at T > 80 °C. In both cases, it was concluded that increasing temperature caused an increase in the mass flux through the membrane.

For the efficient operation of membrane distillation, it is required to avoid the wetting of the pores, as it causes the reduction of flux and the quality of the permeate [10]. Wetting can arise due to the excess of the Liquid Entry Pressure (LEP). Additionally, fouling in the membrane can further promote wetting, as the LEP gets reduced [10,11]. LEP is an essential parameter for the operation of porous hydrophobic membranes: it is defined as the highest applied transmembrane hydrostatic pressure before the liquid in the feed penetrates the larger pores and thus migrates through the hydrophobic membrane [12]. When the transmembrane pressure is kept below the LEP, the liquid in the feed does not penetrate the pores and a liquid-vapor interface is formed at the pore entrance so that only the diffusion of the vapor phase takes place [13]. Only a few studies have investigated the influence of temperature and system pressure on the Liquid Entry Pressure: García-Payo et al. [14] and Guillen-Burrieza et al. [15] experimentally investigated LEP up to 60 °C, Saffarini et al. [16] until 70 °C and Varela-Corredor et al. [17] characterized the LEP of titania-based tubular membranes up to 105 °C. They also measured a liquid entry temperature of nearly 130 °C by establishing a very low constant transmembrane pressure.

The purpose of this study is to experimentally investigate the influence of higher temperatures and increased pressures on the LEP on different membranes.

## 2. Liquid Entry Pressure

### 2.1. Theoretical Principles

**Figure 1:**
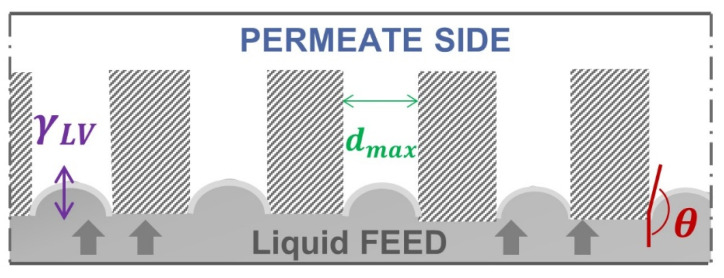
Parameters influencing the Young-Laplace equation on a membrane with ideal cylindrical pores.

The Liquid Entry Pressure can be calculated based on the Young–Laplace equation [12]. According to Equation (1) and Figure 1, the LEP depends on the surface tension of the liquid $\gamma_{LV}$**,** the largest size $d_{max}\;$ and geometric factor ***B*** of the membrane pores and the contact angle of the liquid at the pore entrance (θ):

(1) $$LEP=\Delta p=p_{Feed}-p_{permeate}=\frac{-4\cdot \;B\cdot \;\gamma_{LV}\cdot \;cos\theta }{d_{max}\;}$$

The liquid surface tension here described as the capillary force responsible for forming a convex meniscus in the membrane pore that prevents liquid breakthrough [11]. An increase in temperature, and therefore, in molecular thermal activity, results in the decrease of the surface tension, as the cohesive interaction between the phases falls with the temperature [18,19]. Preventing wetting at higher temperatures is, therefore, a difficult task [11].

The equilibrium contact angle is defined in this context as the interaction between the liquid feed and the solid membrane surface [20]. For proper operation of hydrophobic membranes, a contact angle of around 130° is recommended in the literature [20]. The process conditions, as well as the concentration of the solutes in the fluid in contact with the membrane may have a certain impact on the contact angle and the surface tension, and thus on the LEP [20]. For example, Ge et al. [21] studied the influence of wetting with the temperature measuring the contact angle at 55 °C and 77 °C and observed a decrease in contact angle.

The largest pore size of the membrane plays a crucial role to avoid membrane wetting; therefore, a maximum pore size of 0.6 μm [7] with a uniform pore size distribution is recommended in the literature. The dimensionless parameter ***B*** considers the geometry of the pores. For ***B*** = 1 pores are assumed to be cylindrical. For ***B*** values between 0 and 1, other pore forms are considered. However, since in a real membrane the pores may not be cylindrical nor parallel and may present diverse geometries and be cross-linked [16], there exists no direct correlation of the shape and the value of ***B***. Furthermore, the variation of shapes in a real membrane will also play a role in the determination of the value ***B***. It acts more as an adaptable parameter. Special attention has been made over the years in the proper modeling of pore geometry, pore length, and size. A complete review of the models can be found in the works of Rezaei [11] and Chamani [10]. However, models considering the temperature influence on the LEP are still missing. 

### 2.2. Experimental Determination

Despite the availability of models in the literature, no single model was found to exhibit good predictability for all membrane types [22,23,24]. Hence, the modeling of the LEP serves as a guideline for the operation of the membrane, but the experimental investigation under plant operating conditions [6] remains unavoidable. Real factors which may lead to a lower LEP than predicted, like defects in the geometry or in the hydrophobic coating, larger pores than expected, etc., are not covered in the models. The four main experimental methods are shown in Table 1.

## 3. Materials and Methods

### 3.1. Materials

Different commercial types of flat sheet hydrophobic polymeric and ceramic membranes were used in this study:

- PEEK with a mean pore size of 100 nm (active layer) was acquired from Novamem Ltd. (Schlieren, Switzerland).
- Versapor acrylcopolymer membranes were provided by Pall (US), with 200 nm as mean pore size respectively. The support structure of these Versapor membranes is made of Nylon.
- PTFE membranes were provided by Parker (US). On the one side, Aspire-QP955 and Aspire-QP944C with PET Support and pore sizes of 100 nm and 200 nm respectively. On the other side, the Aspire QL217 with pore sizes of 200 nm and with a support layer made of polypropylene.
- Ceramic membranes were acquired at Fraunhofer-IKTS (Germany). These ceramic membranes are made of Alumina α-Al_2_O_3_ and are asymmetrically built: a porous substrate with a mean pore size of 2.5 µm and the active layer, with pore sizes of 100, 200, and 400 nm respectively. The active layer was modified by a hydrophobic agent resistant to operating temperatures of at least, 230 °C.

Further information provided by the manufacturer can be found in Table 2. 

### 3.2. Methods

#### 3.2.1. Membrane Pre-Characterization

All the membranes were examined by scanning electron microscopy as received. A JXA-8530F Field Emission Electron Probe Microanalyzer (JEOL Ltd., Tokyo, Japan) operating at 5 keV was used for the characterization of the internal morphology. Samples were attached with conductive carbon pads and additionally coated with a conductive carbon layer. Static contact angle measurements were done manually with deionized water with an optical contact angle measurement system OCA5 (DataPhysics Instruments GmbH, Filderstadt, Germany). 

#### 3.2.2. Determination of LEP—Visual Method 

For the determination of the liquid entry pressure using the visual method, a special testing device based on [41] consisting of two Poly (methyl methacrylate)-PMMA plates was used. The membrane samples, with a common area of 10 × 20 mm², were clamped in the PMMA test cell. Depending on the membrane thickness, a spacer of 1 or 2 mm was necessary on the permeate side to ensure tightness. One silicone flat gasket specially optimized for the membranes was used to seal the feed side of the membrane. The gasket allowed for a testing area of 5 mm², corresponding to a 5 mm long and 1 mm wide channel. The transparency of the test cell material was advantageous for water breakthrough detection. 

**Figure 2:**
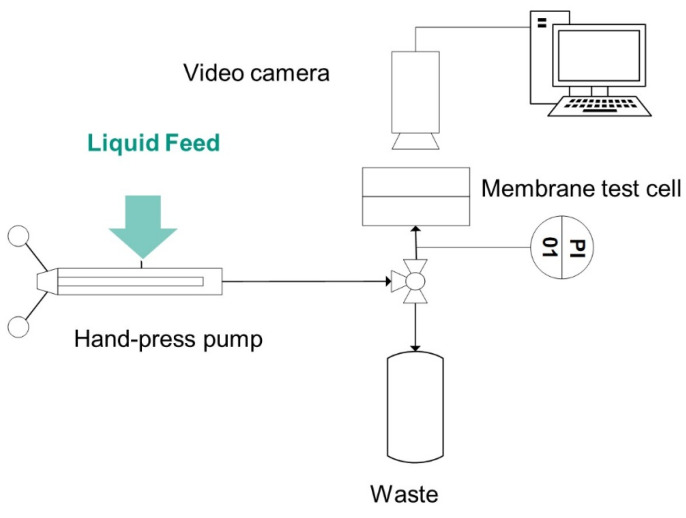
Overview of the experimental set-up used to measure the LEP using the Visual Method.

Figure 2 shows schematically the test rig. To conduct the feed liquid towards the membrane, and precisely set the pressure in the feed chamber, a manually operated SITEC manual screw press (SITEC-Sieber Engineering AG, Maur, Switzerland) was used. The pressure was increased 0.1 bar each step and held for 30 s. The pressure at which a water droplet was visible through a USB digital microscope on the back side of the membrane was recorded using a pressure sensor (Model P-31, accuracy of 0.005 bar, WIKA Alexander Wiegand SE & Co. KG, Klingenberg, Germany). A three-way valve (DWV-1) ensured the elimination of possible air bubbles in the system. The remaining air pockets could be detected through a transparent inlet channel. 

#### 3.2.3. Determination of LEP—Pressure Step Method 

**Figure 3:**
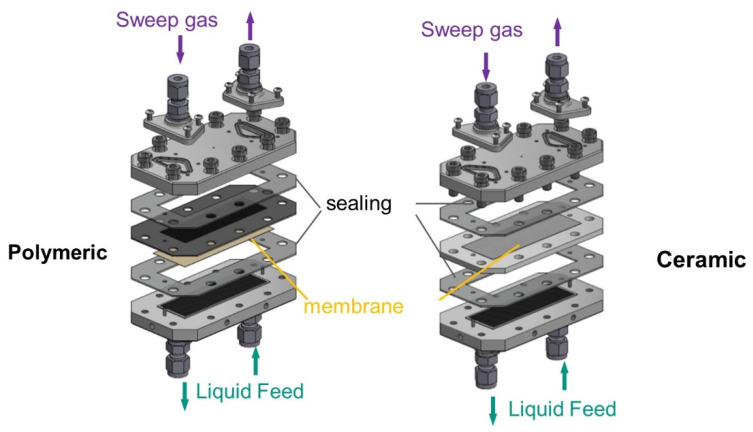
Aluminum test cell used for the LEP pressurized tests; (**Left**) configuration for polymeric membranes; (**Right**) configuration for ceramic membranes.

To be able to carry out measurements of liquid entry pressure at higher temperatures and pressures, a multi-purpose aluminum membrane-test-cell was used, see Figure 3. The test cell allows the testing of different membrane thicknesses and materials as well as the realization of not only LEP measurements but also permeation tests. This membrane test cell is not optically accessible, therefore, the Pressure Step Method was used to detect the LEP [41]. The aluminium membrane-test-cell consists of a base plate with 24 channels of 0.5 × 0.5 mm cross-section as a feed side. The cover plate for the sweep gas side presents 12 channels of 1.5 × 1 mm cross-section. Flat gaskets made of silicon or Viton on each side ensure tightness. Flat sheet membranes with sizes of 31 mm × 87 mm were tested. To test the polymeric membranes, a metallic grid support was used to ensure mechanical stability. For the ceramic membranes, a membrane holder made of PTFE was used. Membrane holders of different thicknesses were used, depending on whether a sintered stainless steel support was used for higher pressure experiments. 

**Figure 4:**
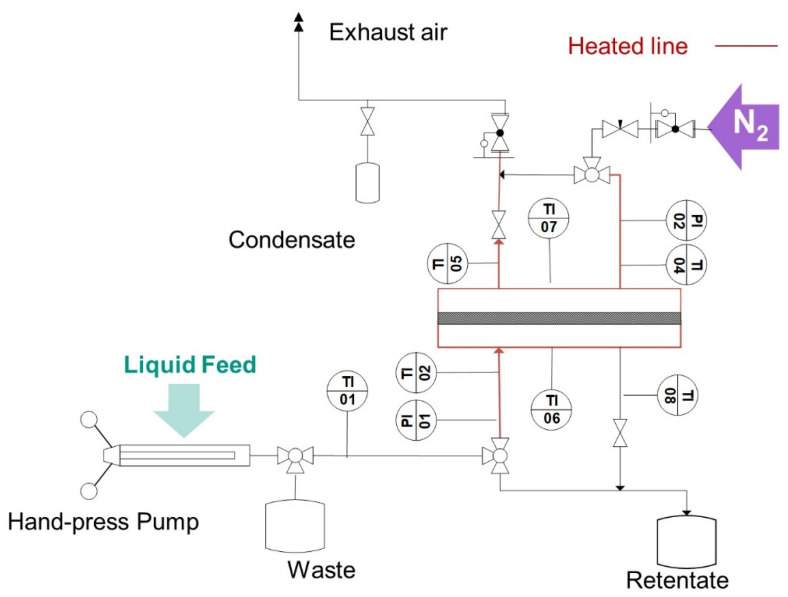
Overview of the experimental set-up used to measure the LEP using the Pressure Step Method.

Electrical heating on the inlet and outlet lines of the test cell, as well as in the test itself (in Figure 4 marked in red) was used to heat the fluid and keep the temperature of the fluid in the cell. Near isothermal experiments were ensured, giving enough time for heating up the fluids and the test cell to reach thermal equilibrium before performing the tests. The temperature was monitored using several thermocouples placed at the inlets and outlets of the gas (TI 04 and TI 05) and water side (TI 02 and TI 08), as well as in the upper and lower side of the test cell shell (TI 06 and TI 07). The pressure in the feed side was built up statically by closing the feed outlet valve and delivering fluid with a SITEC manual screw press (SITEC-Sieber Engineering AG, Maur, Switzerland. To build up the pressure on the permeate side; pressurized nitrogen in counter-current was used. 

The LEP was determined as per the pressure difference detected by two pressure sensors: The pressure sensor (Model P-31, accuracy of 0.005 bar, WIKA Alexander Wiegand SE & Co. KG, Klingenberg, Germany) in the feed side (PI 01) and a second pressure sensor (Model P-30, accuracy of 0.005 bar, WIKA Alexander Wiegand SE & Co. KG, Klingenberg, Germany) in the permeate side. Therewith, the detection of LEP was possible when pressurized experiments with nitrogen on the permeate side were performed. Before each measurement, water was pumped through the system to flush out the air trapped by the assembly of the cell. The pressure was increased in 0.1 bar steps and then held for 12 s. A decrease of the gradient higher than 0.05 bar/s during the holding phases indicated the attainment of the water breakthrough pressure. 

## 4. Results and Discussion

The membranes were characterized under the microscope to characterize their morphological pore structure. Furthermore, to study the behavior of the Liquid Entry Pressure of various membranes in different conditions, experiments were performed first using the Visual Method at ambient conditions. These results were compared with the Pressure Step Method. Next, the influence of increased pressures and temperatures was studied. Within this scope, LEP experiments at room temperature and pressurized conditions were carried out as well as tests at ambient pressure at an increased temperature range (<80 °C). Finally, experiments at the higher temperature range (>80 °C) and an increased pressure were performed.

### 4.1. Morphological Observations

**Figure 5:**
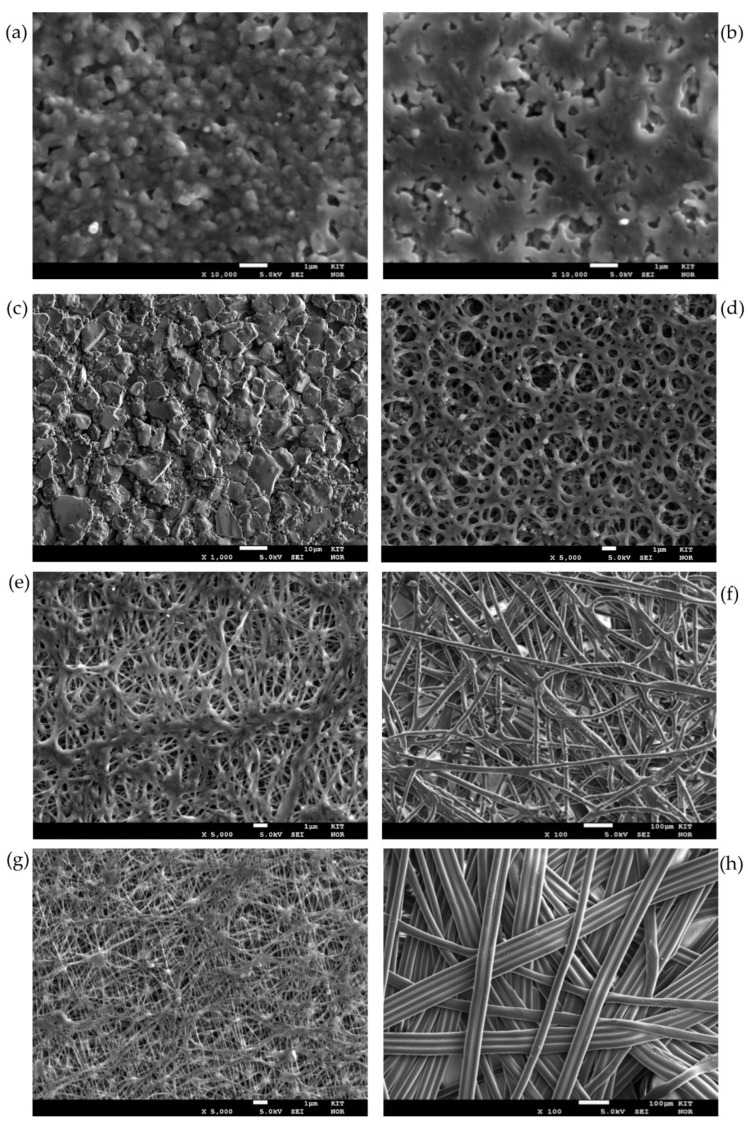
SEM Pictures of various membranes (**a**) PEEK front side (**b**) PEEK back side (**c**) Ceramic 200 front side (**d**) Acrylic Copolymer 200 front side (**e**) PTFE (PET) front side (**f**) PTFE (PET) back side (**g**) PTFE (PP) front side (**h**) PTFE (PP) back side.

Figure 5 shows SEM pictures of the front and/or back sides of the membranes investigated. Figure 5a,b display the front and back sides of the PEEK membrane respectively. A sponge-like structure with cavities as the interconnection of the pores can be distinguished with a flat front membrane surface and a rough back membrane surface. In the case of the ceramic membranes, the pores are the result of the voids between the sintered alumina grains. In this case, the front membrane surface is impregnated by the hydrophobic agent (see Figure 5c). The support displays larger hollow volumes. The Versapor^®^ membrane (acrylic copolymer) displays a trabecular-like meshwork on both sides with the same structure and size (see the front side in Figure 5d). For the PTFE membranes, the selective layer (top layer) has a reticular structure showing interconnected, continuous threads linked by junctions of variable size and morphology (see Figure 5f,e)). The support layer, however, is a nonwoven support, with a much more open structure than the top layer. The difference in support can be seen in Figure 5g (PET Support) and Figure 5h (PP support). 

As it is evident from Figure 5, depending on the membrane material, the pore geometry is different. Even for the same membrane materials, like in Figure 5e,g for the PTFE membranes, the top layer is built up from a random orientation of very thin fibers. This, of course, results in highly variable pore geometries for the same membrane material so that the applicability of a style value for the geometrical parameter ***B*** falls short. It is obvious that the value should be far below 1. 

### 4.2. Liquid Entry Pressure at Ambient Conditions—Visual Method

**Figure 6:**
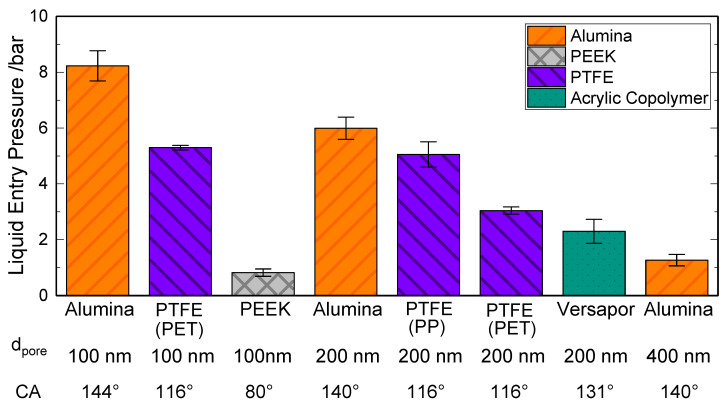
Results of LEP measured by the visual method at ambient conditions T = 25 °C, *p* = 1 bar together with the mean pore size of the membrane (d_pore_) and the measured static contact angle values (CA).

Figure 6 shows the experimental results of the LEP measurement for the membranes presented in Section 2.1, using the experimental set-up explained in Section 3.2, together with the measured values for the static contact angle of deionized water. The values shown in Figure 6 are the average values of at least three consecutive measurements. 

As per Figure 6, the strong dependence of the LEP with the characteristic pore size can be confirmed. In this case, the characteristic pore size is taken as the mean pore size given by the manufacturer, as the largest pore size could not be determined within the current study. It can be observed that membranes with higher characteristic pore sizes, in this case, the Alumina-400, present the lowest LEP which follows the behavior depicted by the Young–Laplace Equation (Equation (1)). It is noteworthy that porous PEEK membranes, promoted as appropriate for membrane distillation, display a contact angle of 80° and still a LEP value of 0.82 bar. 

The results in Figure 6 reveal that for the same characteristic pore size, ceramic membranes present a higher liquid entry pressure than PTFE membranes. For example, in the case of the 100 nm membranes, the ceramic membrane shows a LEP of 8.2 bar (±0.5 bar) and the PET-supported PTFE membrane shows 5.3 bar (±0.08 bar). The reason for this difference is not only the higher hydrophobicity of the ceramic surface area -confirmed by the measured contact angle value of 144° vs. 116°—but also the difference in internal geometry, as already seen in Figure 5. The higher thickness of the tested ceramic membranes (2 mm) in comparison to the PTFE membranes (0.15 mm–0.25 mm) plays an important role, especially when the LEP is detected using the Visual Method. Here, a certain amount of time is required until a drop is formed in the back side of the membrane and is large enough to be visible for the human eye. This positive effect of the membrane thickness on the LEP is also confirmed by the experimental and simulative observations of Guillen-Burrieza et al. [22] and Chamani et al. [42]. Moreover, the possible narrower pore size distribution of ceramic membranes in comparison to polymeric membranes may be a strong factor in this difference. The same findings can be seen for the 200 nm ceramic and PTFE membranes. 

Moreover, in the case of the 200 nm PTFE membranes, a difference in LEP between the PP-supported PTFE membranes and the PET-supported membranes can be observed in Figure 6. The reason for this difference might be, apart from a possible different pore size distribution, the fact that PP is naturally hydrophobic and PET is naturally hydrophilic. Thus, leading to a faster formation of visible drops in the back side of the PET-supported membrane. The morphological differences between the support can be observed in Figure 5. Besides, for the acrylic copolymeric membrane, even if showing a higher surface hydrophobicity in terms of contact angle (i.e., 131°), a lower LEP value was observed (2.3 bar + −0.4 bar). The reason here may also be the influence of the internal membrane morphology on the LEP. The role of the membrane morphology on the LEP has also been studied by Saffarini [16].

### 4.3. Liquid Entry Pressure at Ambient Conditions—Pressure Step Method

**Figure 7:**
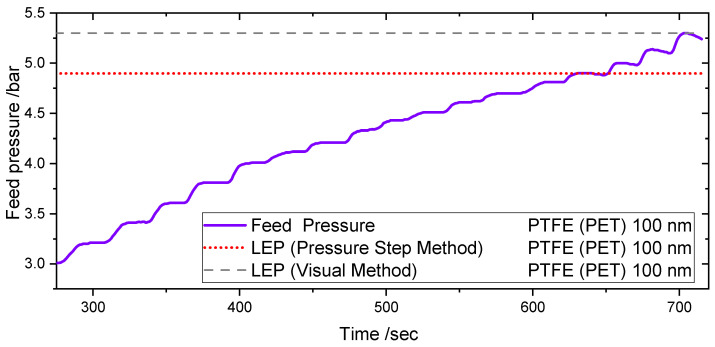
Pressure course during LEP detection using Pressure Step Detection in the PMMA test cell. The pressure decrease during the steps shows the achievement of the Liquid entry Pressure.

Figure 7 shows the pressure course during one LEP test using the Pressure Step Method and the Visual Method in the PMMA test cell for a 100 nm PTFE (PET) membrane at ambient conditions. The experiments were performed at room temperature and atmospheric pressure. 

When 4.8 bars are reached in the feed side, a negative pressure gradient can be observed as a result of the water leakage through the membrane. Comparing the value with the value obtained within the visual method, the value is 10% lower. The difference in results leads to the conclusion that the Pressure Step Method is an early detection method of the LEP, in comparison to the Visual Method for the same cell and operating conditions.

**Figure 8:**
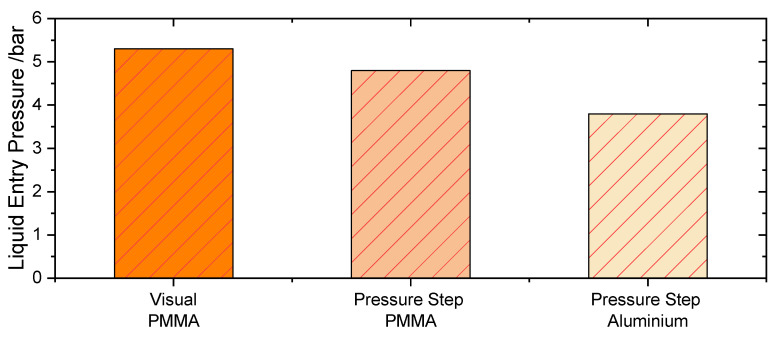
LEP values for the PTFE (PET) 100 nm membrane using the Visual Method and the Pressure Step Method in the PMMA cell as well as the Pressure Step Method results in the aluminum test cell.

Furthermore, Figure 8 shows the comparison of the LEP value obtained in the PMMA test cell via the Visual Method and Pressure Step Method as well as the results of the Pressure Step Method in the aluminum test cell at room temperature and atmospheric pressure. Thus, without nitrogen flowing in the permeate side of the aluminum test cell. Here a further reduced LEP can be observed. The differences in Figure 8 are not only due to the different detection methods but also are influenced by the difference in the tested area. In the case of the Pressure Step Method in the aluminum test cell, a membrane area of 2697 mm² is tested, instead of the 5 mm² test membrane area within the PMMA cell. This difference in test area may lead to a higher probability of having larger-than-average pores in the alumina test cell, and therefore, lower LEP might be obtained. 

Overall, the characterization of the LEP in the same cell where further permeation experiments are going to be performed (in this case, the aluminum test cell) leads to a more accurate characterization of the membrane for the application. These findings are in agreement with the results of Warsinger [41], as well as the comparison made by Racz [20] between the dynamic and static methods [10].

### 4.4. Study of the Liquid Entry Pressure at Higher Temperatures and Pressures 

#### 4.4.1. Influence of Higher System Pressures

To study the influence of increased pressure on the LEP, measurements at room temperature and system pressures up to 2.5 bars for pure water were performed. The permeate side was pressurized by nitrogen gas. The pressure increase was done in a stepwise manner, to ensure that both sides of the membrane are pressurized to the same extent. During all tests, special care was taken to ensure that the water pressure was always slightly higher than the gas pressure so that no nitrogen flows through the membrane to the feed side. When the desired system pressure was reached, the feed pressure was further increased until the LEP could be detected. 

**Figure 9:**
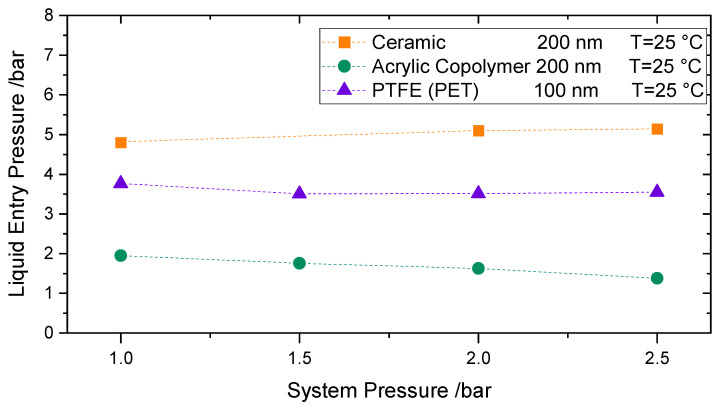
Influence of system pressures up to 2.5 bar abs on Liquid Entry Pressure for an Acrylic Copolymeric Membrane 200 nm (Green), Ceramic Membrane 200 nm (Orange), and a PTFE (PET) membrane 100 nm (100).

Figure 9 shows the LEP results for 3 different membranes at room temperature: the acrylic copolymeric membrane-200 nm, the alumina membrane-200 nm, and the PTFE membrane supported by PET-100 nm. Each point is the average of two consecutive measurements. As per Figure 9, no trend can be identified when system pressure is increased; the LEP looks to remain barely unaffected. This behavior can be explained by the minor effect of the pressure in the surface tension in the studied range, where the intermolecular forces between liquid molecules remain nearly unchanged [43]. Accordingly, no remarkable effect can be observed in the measured LEP. Based on [44,45], when increasing the pressure up to 2.5 bars, only a 0.3% decrease in surface tension can be expected [6,46,47,48]. The slight differences in LEP behavior for the different membranes may be due to secondary effects occurring during the measurement.

#### 4.4.2. Influence of Higher System Temperatures (T < 80 °C)

**Figure 10:**
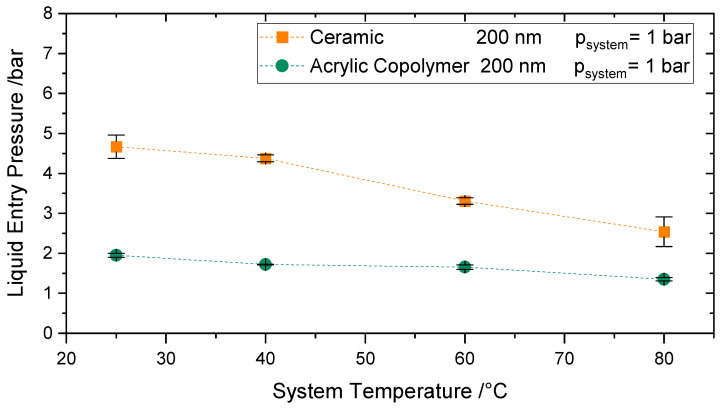
Influence of system temperatures up to 80 °C on Liquid Entry Pressure for a Versapor Membrane 200 (Green) and Ceramic Membrane 200 (Orange).

The effect of temperatures up to 80 °C at atmospheric pressure on the LEP was investigated for the Versapor^®^ 200-R and the ceramic membrane IKTS-200, both with a reported pore size of 200 nm. Figure 10 reveals a decrease of 30% (for the Versapor Membranes) and of 45% (for the ceramic membranes) of LEP for temperatures up to 80 °C. At ambient pressure, a decrease of 13% [48] in surface tension is expected when the temperature is increased up to 80 °C, see. The remaining LEP decrease at this temperature range (15–30%) may be due to the change of contact angle with temperature and further secondary effects.

The decrease of LEP up to 50 °C is in agreement with the LEP behavior reported by Garcia Payo et al. [14] in the same temperature range for a PVDF22 membrane, i.e., a 13% decrease in comparison to ambient conditions. The decrease of LEP up to 70 °C is 7% larger than the LEP decrease measured by Saffarini et al. [16] at the same temperature range for PTFE membranes. Saffarini et al. accounted for this decrease not only due to the decrease in surface tension and contact angle but also due to the alteration of the membrane microstructure with temperature. These comparisons should be taken with care, as even if the fluid and the conditions may be equivalent, membranes here are made of different materials.

#### 4.4.3. Influence of Higher System Temperatures (T > 80 °C) 

**Figure 11:**
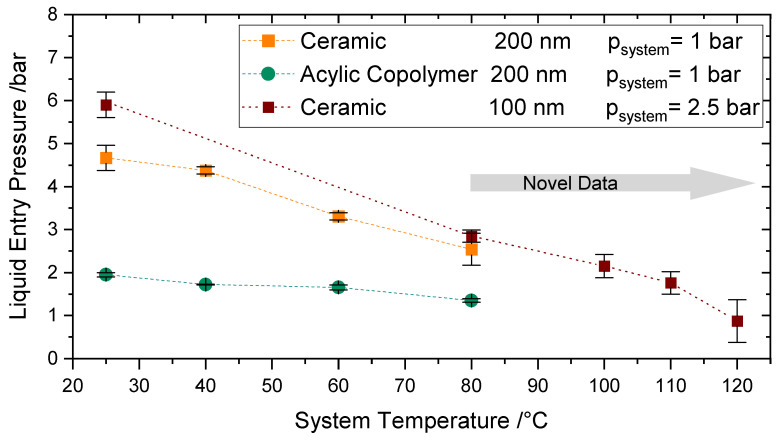
Influence of system temperatures up to 120 °C on Liquid Entry Pressure for Ceramic Membrane 100 (Red) at a system pressure of 2.5 bar compared to the Liquid Entry Pressure for a Versapor Membrane 200 (Green) and Ceramic Membrane 200 (Orange) up to 80 °C.

The measurements of LEP at temperatures between 80–120 °C were performed by raising the system pressure to a value above the corresponding saturation pressure of the operating temperatures to avoid boiling. The pressure and temperature were increased alternately to avoid the boiling of water and the flow of nitrogen into the feed compartment. A further decrease of LEP due to the temperature increase up to 120 °C can be observed in Figure 11 in comparison to Figure 10, where the experiments were performed up to 80 °C. 

As already confirmed in Section 4.4.2, the effect of the system pressure in the surface tension is unremarkable; therefore, the decrease in LEP is mainly a result of the temperature increase. At 120 °C a value of 0.9 bar was measured. A LEP of at least 2.5 bars is recommended in the literature [49] to avoid wetting. However, membranes with lower LEP values may be applicable if the membrane module or reactor is accordingly designed so that the transmembrane pressure never exceeds the LEP. This can be achieved by considering the right pressure, volume flow rate, and hydraulic diameter of the feed and permeate side.

To the best of the authors’ knowledge, there exist no comparable values of LEP directly measured at constant temperature conditions in the literature at this higher temperature range and increased pressure. Varela-Corredor [17] characterized the LEP of titania-based tubular membranes up to 105 °C. He measured a liquid entry temperature of nearly 130 °C by establishing a very low constant transmembrane pressure. 

Altogether, being able to measure directly LEP at the temperature region considered in the present work broadens the application range of hydrophobic membranes and enables the use of the membranes for unexplored fields. These results are especially relevant for the application of these membranes for Sweep Gas Membrane Distillation, as the operating parameters can be reproduced.

## 5. Conclusions

The determination of LEP using mathematical models is only possible for pre-defined membrane morphologies. From the literature, four different experimental methods to measure the LEP have been identified. Within the scope, experiments were carried out to characterize different hydrophobic membranes using the Visual Method and the Pressure Step Method.

It was confirmed that the (largest) pore size of the membrane is a crucial parameter on the LEP. Results revealed that the tested ceramic membranes have higher LEP which may be due to not only higher contact angle of the surface, but also a higher thickness and narrower pore size distribution than the PTFE membranes. The comparative results between the Visual Method and the Pressure Step Method in the same cell confirmed the Pressure Step Method as an earlier detection method. Besides, it was confirmed that it is crucial to characterize the membranes in the test cell that will be used for the operation of the membrane.

The tests at increased system pressure revealed a negligible influence of pressure of LEP due to its minor effect on the surface tension at the studied pressure range. The tests of LEP performed at increased temperatures, showed a stronger decrease of LEP, revealing the great influence of temperature on the surface tension and in turn of the LEP. The novel data generated at temperature up to 120 °C indicates a further decrease of LEP irrespective of the pressurization degree.

Within this study, the highest liquid entry pressure was measured for the alumina membranes. In addition, the high thermal stability of the ceramic material and high contact angles of the hydrophobized surface, make this sort of ceramic membrane best suited for sweeping gas membrane distillation at temperatures above 80 °C. A metal support structure is required, however.

The results showed in this study provide very valuable information to ensure the reliable performance of separation experiments to remove steam effectively up to temperatures of at least 120 °C when the transmembrane pressure is kept below the measured LEP.

Further LEP characterization tests are required when organic compounds are present in the feed, as the LEP may be negatively affected as a result of the surface tension decrease. When organic compounds are present in the feed, the LEP may be negatively affected as a result of the surface tension decrease. For this reason, further LEP characterization tests are recommended when dealing with these feed compositions.

## Figures and Tables

**Table 1:** Overview of experimental methods to determine the Liquid Entry Pressure in the literature.

Measurement Method	Measurement Principle	Studies	Advantages	Disadvantages
Visual	Pressure at which the first water drop can be visually detected on the back side of the membrane	[14,15,16,20,22,24,25,26,27,28,29,30,31,32,33]	Simple setup Frequent use	Subjective method The membrane needs to be visually accessible Experiments at higher temperatures and pressures are not easily possible
Conductivity	Detection of an increase in conductivity in the permeate side	[20,22,34,35,36,37]	Fast detection of liquid breakthrough	Only suitable for electrically conducting solutions
Pressure Step	The gradient dp_feed_/dt becomes negative during the increase of the feed pressure	[38]	Fast detection of liquid breakthrough Non-dependent on the feed composition	Very accurate and rapid pressure sensors are required
Flow curve (hysteresis)	Pressure at which the flow rate vs. transmembrane pressure reaches a threshold value	[13,14,17,20,39,40]	Potential detection of defects in the membrane Accurate detection of breakthrough when membrane parameters are known	Very accurate and pressure and flow sensors are required The threshold value should be specified Previous accurate pore characteristics (tortuosity, pore size distribution, porosity) determination required

**Table 2:** Membrane parameters given by manufacturers.

Membrane Name	Material	Support	Pore Size (nm)	Total Thickness (mm)	LEP (bar)
PEEK100	PEEK	PEEK	100	0.03–0.04	-
Versapor^®^ 200-R	Acrylcopolymer	Nylon	200	0.15–0.20	1.79
Aspire^®^-QP955	PTFE	PET	100	0.15–0.25	>4.5
Aspire^®^-QP944C	PTFE	PET	200	0.08–0.18	>2.3
Aspire^®^-QL217	PTFE	PP	200	0.15–0.25	>1.0
IKTS-100	Hydrophobized α-Al_2_O_3_	Alumina	100	2	-
IKTS-200	Hydrophobized α-Al_2_O_3_	Alumina	200	2	-
IKTS-400	Hydrophobized α-Al_2_O_3_	Alumina	400	2	-

Deionized water was used as feed liquid. Nitrogen in the sweep gas side was used for the experiments.

## Data Availability

Not applicable.
